# Retrospective Comparative Analysis of KRAS G12C *vs*. Other KRAS Mutations in mCRC Patients Treated With First-Line Chemotherapy Doublet + Bevacizumab

**DOI:** 10.3389/fonc.2021.736104

**Published:** 2021-09-30

**Authors:** Riccardo Giampieri, Alessio Lupi, Pina Ziranu, Alessandro Bittoni, Andrea Pretta, Federica Pecci, Mara Persano, Enrica Giglio, Cecilia Copparoni, Sonia Crocetti, Alessandra Mandolesi, Gavino Faa, Pierpaolo Coni, Mario Scartozzi, Rossana Berardi

**Affiliations:** ^1^ Clinica Oncologica—Dipartimento Scienze Cliniche e Molecolari—Università Politecnica delle Marche, Ancona, Italy; ^2^ Clinica Oncologica—Azienda Ospedaliera Universitaria Ospedali Riuniti di Ancona, Ancona, Italy; ^3^ Oncologia, Università ed Azienda Ospedaliera Universitaria di Cagliari, Cagliari, Italy; ^4^ Oncologia Medica, Università “la Sapienza” di Roma, Rome, Italy; ^5^ Anatomia Patologica—Azienda Ospedaliera Universitaria Ospedali Riuniti di Ancona, Ancona, Italy; ^6^ Anatomia Patologica—Dipartimento di Scienze Mediche e Sanità Pubblica—Università di Cagliari, Cagliari, Italy

**Keywords:** mCRC, KRAS, G12C, first line, chemotherapy

## Abstract

**Background:**

KRAS mutations in metastatic colorectal cancer (mCRC) define a subset of tumors that have primary resistance to anti-EGFR-based therapy. Data concerning whether different KRAS mutations may also have a prognostic value are lacking. Furthermore, novel KRAS G12C inhibitors are currently in development. The aim of our analysis was to compare response rates in patients treated with first-line chemotherapy doublet + Bevacizumab among different KRAS variants. Secondary end-points were progression free survival (PFS) and overall survival (OS).

**Methods:**

Patients with KRAS mutated mCRC treated with either FOLFIRI/FOLFOX/XELOX + Bevacizumab were eligible for enrollment. Patients whose tumor harbored NRAS mutations or that coexpressed also BRAF mutations were excluded from this retrospective analysis. Patients’ individual data were collected from patients’ records. Propensity score matching (nearest method, 1:2 ratio) was used to define the two different groups of patients for comparison (KRAS G12C mutated *vs* other KRAS variants). Eastern Cooperative Oncology Group Performance Status (ECOG PS), sex, metastatic site of involvement, synchronous *vs* metachronous metastatic disease, tumor sidedness, mucinous histology, primary tumor surgery, more than two lines of treatment for metastatic disease, and radical surgery of metastases were used as matching factors. Response rate (RR) was calculated by RECIST 1.1 criteria. Both progression free-survival and overall survival were calculated by Kaplan–Meier method. Categorical variables were compared by Fisher exact test for binomial variables and by chi-square test for all other instances. The level of statistical significance p was set at 0.05 for all tests.

**Results:**

A total of 120 patients were assessed in the final analysis. Out of the 120 patients, 15 (12%) were KRAS G12C mutated. In the whole cohort of patients, 59/120 (49%) had partial response (PR), 42/120 (35%) had stable disease (SD), and 19/120 (16%) had progressive disease (PD) as the best response. In KRAS G12C patients, 4/15 (27%) had PR, 6/15 (40%) had SD, and the remaining 5/15 (33%) had PD as the best response. In patients with other KRAS mutations, 55/105 (52%) had PR, 37/105 (35%) had SD, and the remaining 13/105 (12%) had PD as the best response. The difference in RR between the two groups of patients was statistically significant (p=0.017). On the other hand, no difference in PFS (p=0.76) and OS (p=0.56) was observed. After matching procedures, the difference in response rates between KRAS G12C mutated patients *vs* the matched cohort of patients with other KRAS mutations remained statistically significant (p=0.016). KRAS G12C mutations were not associated with differences in sites of metastatic involvement, sex, and ECOG PS. On the other hand, synchronous *vs* metachronous metastatic disease (p=0.039), age > 75 years (p=0.043), and mucinous histology (p=0.008) were more frequent in G12C mutated tumors.

**Conclusions:**

In our cohort of patients, it was observed that KRAS G12C mutations are associated with worse response rates compared to other KRAS variants when treated with standard chemotherapy doublet + Bevacizumab. On the other hand, both PFS and OS were not significantly different. Based on these findings, we believe that new treatment options focused on KRAS G12C inhibition should be tested mainly in first-line setting and in addition to standard chemotherapy doublet + Bevacizumab for mCRC patients, as they might “fill the gap” in response rates that was seen in our study.

## Background

In the last decade, molecular evaluation in patients with metastatic colorectal cancer (mCRC) has been widely used to assess primary resistance to anti-epidermal growth factor receptor (EGFR) based therapy.

Retrospective studies conducted in anti-EGFR monotherapy trials have shown that Kirsten Rat Sarcoma viral oncogene homologue (KRAS) mutations confer resistance to anti-EGFR-based therapy (1, 2). These initial reports were confirmed also in CRYSTAL (3) and PRIME (4) trials where KRAS mutated status was associated with resistance to anti-EGFR drugs even when combined with standard first-line palliative chemotherapy.

Similar results were retrospectively observed in all trials where anti-EGFR were used for mCRC patients: because of this fact, KRAS and Neuroblastoma RAS Viral oncogene homologue (NRAS) wild-type status is mandatory for treatment with either Panitumumab or Cetuximab in patients with mCRC. Other markers of primary resistance to anti-EGFR-based therapy have been tested (5–7) but have failed to be introduced in standard clinical practice.

While it is well documented that KRAS mutations confer resistance to anti-EGFR treatment, data focused on different clinical behavior of specific KRAS mutations are, indeed, lacking. Part of the explanation of this problem might be traced back to different distribution of various KRAS mutations across various tumor types: in mCRC for example, KRAS G12D, G12V, and G12A are frequently observed, whereas G12C mutation accounts only for 9–10% of all KRAS mutations. On the other hand, in non-small cell lung cancer (NSCLC), KRAS G12C mutations are highly prevalent (40% of all KRAS mutations). In pancreatic cancer, G12D mutation is the most frequent along with G12V, G12C mutations are rarely seen, and G12R becomes also relatively frequent (while G12R is rarely seen in NSCLC and almost nonexistent in mCRC) (8).

Whether KRAS variants are associated with different clinical behavior is still open debate: from a theoretical point of view, various mutations might be associated with entirely different intracellular signaling pathways, and this fact might cause differences in protein function. When a mutation does occur in KRAS mutational hotspots, it leads to production of constitutively activated proteins, free from the need of upstream tyrosine-kinase receptor associated recruitment; it has been, however, reported that not all KRAS mutations determine similar changes in kinase activity of the protein itself: for example, G12D mutations have been associated with increased catalytic activity of K-ras protein compared to its wild-type counterpart, but this has not been consistently described also for other KRAS mutations (9).

G12C mutation seems to be associated with worse prognosis in patients with resected lung cancer (10). Moreover, in lung cancer patients treated with palliative chemotherapy, KRAS G12C mutation was associated with worse prognosis, while other KRAS variants were not significantly associated with different outcome (11).

In colorectal cancer, the role of G12C mutations, owing to the expected lower frequency of this mutation, is subject to debate. The aim of our analysis was to assess the prognostic impact of the KRAS G12C variant compared with other KRAS mutations in patients with mCRC treated with standard first-line palliative chemotherapy.

## Methods

Patients with KRAS mutated metastatic colorectal cancer, treated with first-line Bevacizumab plus chemotherapy doublet, were enrolled in this retrospective analysis. All patients should have started treatment at full-dose first-line chemotherapy. Further dose reductions due to the onset of toxicity were allowed. Patients were treated in the years 2010–2020 time range. This multicenter analysis included patients treated at Clinica Oncologica AOU Ospedali Riuniti di Ancona (Ancona, Marche, Italy) and at Oncologia Medica, Azienda Ospedaliera Universitaria di Cagliari (Monserrato, Sardegna, Italy). The study was submitted and approved to the local ethics committee before study initiation. The study adheres to the principles of Helsinki Declaration concerning ethical principles in human experimentation.

KRAS mutational status assessment was performed either by pyrosequencing (mainly for patients who were treated before 2017) or by the matrix-assisted laser desorption/ionization time of flight (MALDI-TOF) technique. Both methods are approved by standard national guidelines and have sensitivity threshold around 5%, as required by those guidelines.

Variables that were considered for univariate analysis were sex (male *vs* female), age (< or >75 years old), different sites of metastatic involvement (liver metastases yes *vs* no, lung metastases yes *vs* no, peritoneal metastases yes *vs* no) and timing (metachronous *vs* synchronous), mucinous histology (yes *vs* no) and tumor grade (G1 *vs* G2 *vs* G3), tumor sidedness (right *vs* left-sided), resection of primary tumor (yes *vs* no) and resection of metastases with radical intent (yes *vs* no), Eastern Operative Oncology Group Performance status (ECOG PS) at the start of first-line treatment, and having received other lines of treatment after the first and second line, respectively.

The primary end-point of this analysis was to demonstrate a statistically significant difference in terms of response rate among patients with KRAS G12C *vs* other mutations. Response rates (RRs) were assessed by RECIST 1.1 criteria. Secondary end-points were progression free survival (PFS) and overall survival (OS). Survival outcomes were calculated by the Kaplan–Meier method.

KRAS G12C samples were matched with other KRAS mutations by propensity score matching (“Nearest” method, ratio 1:2) (12). Matching variables included ECOG PS, sex, metastatic site of involvement, synchronous *vs* metachronous metastatic disease, tumor sidedness, mucinous histology, primary tumor surgery, and radical surgery of metastases, having received more than two lines of treatment for metastatic disease.

Binomial categorical variables were assessed by Fisher’s exact test, while other categorical variables were assessed by the chi-square test. Univariate survival analysis among different variables used for stratification was assessed by the log-rank test, whereas Cox proportional-hazard regression was used for multivariate analysis.

The level of statistical significance p was set at 0.05 for all tests.

All analyses were conducted by using the MedCalc Statistical Software version 19.2.1 (MedCalc Software Ltd, Ostend, Belgium; https://www.medcalc.org; 2020) and by R software (version 3.6.2).

## Results

A total of 218 KRAS mutated patients were initially screened. Among them, 120 were treated with first-line chemotherapy doublet + Bevacizumab and were further analyzed. Among the 98 patients who were excluded, 74 received chemotherapy without Bevacizumab, 7 were treated with FOLFOXIRI + Bevacizumab regimen, 4 received monochemotherapy, and the remaining 13 received monochemotherapy + Bevacizumab.

**Figure 1:**
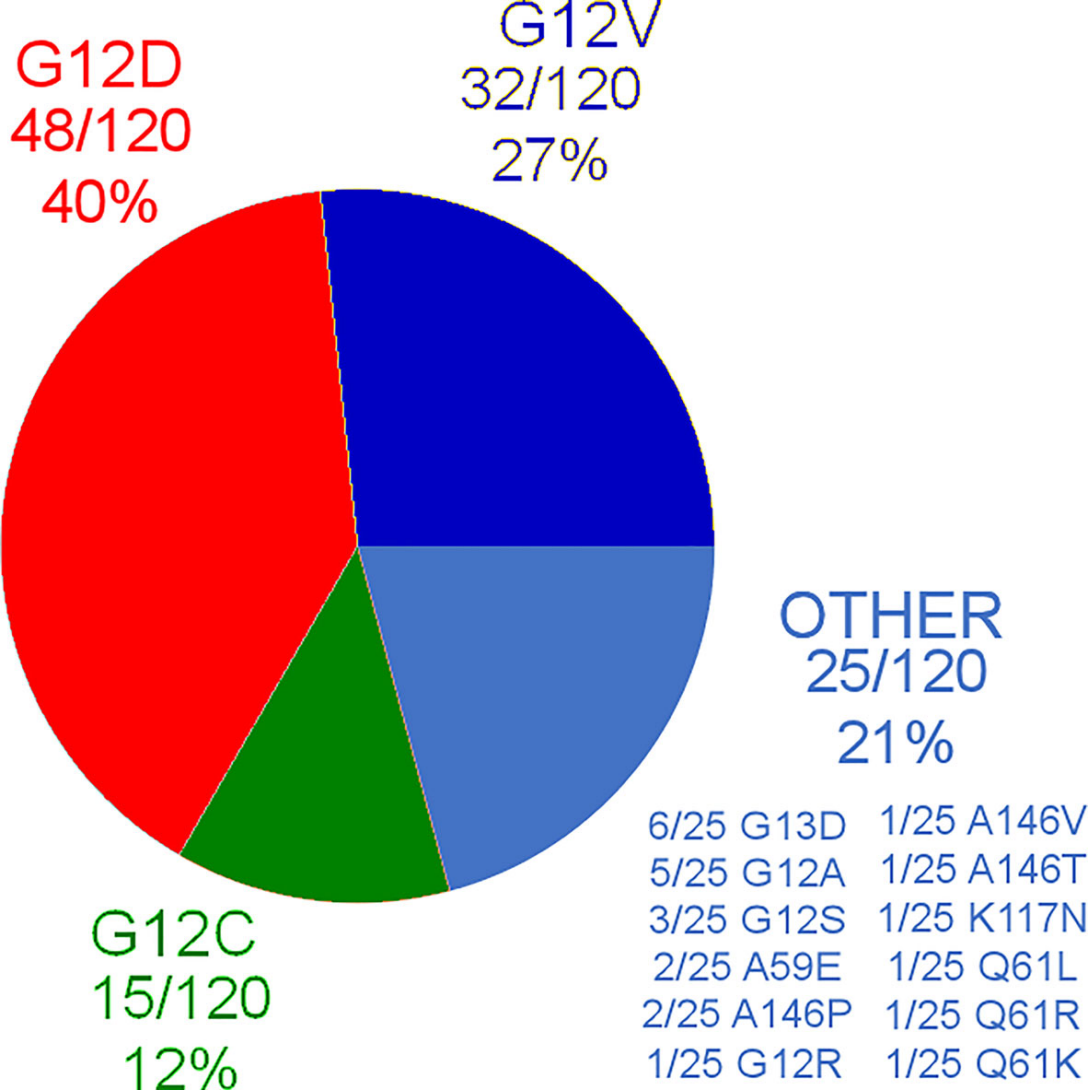
Summary of KRAS mutations in the analyzed cohort.

Out of the 120 patients, 15 (12%) were KRAS G12C mutated, 48/120 (40%) were KRAS G12D mutated, 32/120 (27%) were KRAS G12V mutated, and the remaining 25/120 patients had other KRAS mutations different from G12C, G12D, and G12V. Further distribution of different KRAS mutations can be found in **Figure 1**. As it can be seen, 10/120 patients (8%) carried exon3/4 mutations.

The summary of patients’ clinical characteristics, as well as differences in distribution of the stratification variables among different KRAS variants, can be found in **Table 1**. The only variable that resulted to have differences in distribution among all patients included in the analysis was the presence of lymphovascular invasion in the tumor, which resulted to be more frequently observed in KRAS G12V and other mutations different from either G12C or G12D (p=0.004).

**Table 1:** Patients’ clinical characteristics according to KRAS variants.

Patient’s feature	G12C	G12D		G12V	Other	p value
**Total patients**	15	48		32	25	
**Age at diagnosis**						
<75 years	11 (74%)	46 (96%)		29 (91%)	22 (88%)	0.08
≥75 years	4 (26%)	2 (4%)		3 (9%)	3 (12%)	
**Gender**						
Male	5 (33%)	28 (58%)		21 (66%)	15 (60%)	0.20
Female	10 (67%)	20 (42%)		11 (34%)	10 (40%)	
**ECOG-PS**						
0	14 (93%)	35 (73%)		20 (63%)	21 (84%)	0.13
1	1 (7%)	11 (23%)		12 (37%)	4 (16%)	
2	0 (0%)	2 (4%)		0 (0%)	0 (0%)	
**Lymphovascular/perineural invasion**						
Yes	2 (13%)	30 (62%)		26 (81%)	20 (80%)	**0.0004**
No	10 (67%)	13 (27%)		4 (12%)	3 (12%)	
Unknown	3 (20%)	5 (11%)		2 (7%)	2 (8%)	
**Sidedness**						
Right	7 (47%)	12 (25%)		13 (40%)	11 (44%)	0.06
Left	5 (33%)	20 (42%)		17 (53%)	11 (44%)	
Rectum	3 (20%)	16 (33%)		2 (7%)	3 (12%)	
**Tumor grade**						
1	2 (13%)	3 (6%)		3 (9%)	0 (0%)	0.35
2	11 (73%)	27 (56%)		19 (59%)	21 (84%)	
3	1 (7%)	9 (19%)		5 (16%)	1 (4%)	
Unknown	1 (7%)	9 (19%)		5 (16%)	3 (12%)	
**Mucinous histology**						
Yes	6 (40%)	6 (12%)		6 (19%)	1 (4%)	0.05
No	7 (47%)	40 (84%)		24 (75%)	22 (88%)	
Unknown	2 (13%)	2 (4%)		2 (6%)	2 (8%)	
**MSI state**						
P-MMR	4 (27%)	15 (31%)		5 (16%)	5 (20%)	0.51
D-MMR	1 (7%)	1 (2%)		0 (0%)	1 (4%)	
Unknown	10 (66%)	32 (67%)		27 (84%)	19 (76%)	
**Synchronous/Metachronous**						
Synchronous	4 (27%)	27 (56%)		19 (59%)	12 (48%)	0.16
Metachronous	11 (73%)	21 (44%)		13 (41%)	13 (52%)	
**Liver metastases**						
Yes	11 (73%)	32 (67%)		23 (72%)	17 (68%)	0.94
No	4 (27%)	16 (33%)		9 (28%)	8 (32%)	
**Lung metastases**						
Yes	4 (27%)	20 (42%)		12 (37%)	7 (28%)	0.58
No	11 (73%)	28 (58%)		20 (63%)	18 (72%)	
**Peritoneal metastases**						
Yes	2 (14%)	15 (31%)		6 (19%)	5 (20%)	0.38
No	13 (86%)	33 (69%)		26 (81%)	20 (80%)	
**Other metastatic sites**						
Yes	4 (27%)	15 (31%)		7 (22%)	5 (20%)	0.69
No	11 (73%)	33 (69%)		25 (78%)	20 (80%)	
**Surgery (Primary tumor)**						
Yes	15 (100%)	36 (75%)		25 (78%)	18 (72%)	0.17
No	0 (0%)	12 (25%)		7 (22%)	7 (28%)	
**Surgery (Resectable Metastases)**						
Yes	5 (34%)	12 (25%)		7 (22%)	4 (16%)	0.63
No	10 (66%)	36 (75%)		25 (78%)	21 (84%)	
**Adjuvant chemotherapy**						
Yes	9 (60%)	18 (38%)		11 (34%)	8 (32%)	0.30
No	6 (40%)	30 (62%)		21 (66%)	17 (68%)	
**Type of adjuvant chemotherapy**						
FOLFOX	5 (33%)	9 (19%)		6 (19%)	7 (28%)	0.17
XELOX	2 (13%)	8 (17%)		5 (15%)	0 (0%)	
Mono	2 (13%)	1 (2%)		0 (0%)	1 (6%)	
Unknown/No adjuvant	6 (41%)	30 (62%)		21 (66%)	17 (68%)	
**Recurrence after surgery of resectable metastases**						
Yes	5 (100%)	12 (100%)		6 (86%)	4 (100%)	0.37
No	0 (0%)	0 (0%)		1 (14%)	0 (0%)	
**Type of 1^st^ line chemotherapy**						
FOLFOX/XELOX + Bevacizumab	3 (20%)	22 (46%)		15 (47%)	14 (56%)	0.17
FOLFIRI+Bevacizumab	12 (80%)	26 (54%)		17 (53%)	11 (44%)	
**Best response to 1^st^ line chemotherapy**						
PD	5 (33%)	6 (13%)		3 (9%)	4 (16%)	0.23
SD	6 (40%)	17 (35%)		14 (44%)	6 (24%)	
PR	4 (27%)	25 (52%)		15 (47%)	15 (60%)	
**Maintenance 1^st^ line chemotherapy**						
Yes	5 (34%)	19 (40%)		6 (19%)	12 (48%)	0.11
No	10 (66%)	29 (60%)		26 (81%)	13 (52%)	
**Type of 2^nd^ line therapy**						
FOLFOX/XELOX +/- Bevacizumab	7 (47%)	11 (23%)		7 (22%)	8 (32%)	0.67
FOLFIRI +/- Aflibercept/Bevacizumab	3 (20%)	19 (40%)		11 (34%)	10 (40%)	
Other	1 (6%)	5 (10%)		6 (19%)	2 (8%)	
Unknown/Not yet	4 (27%)	13 (27%)		8 (25%)	5 (20%)	
**ECOG-PS 2^nd^ line**						
0	8 (53%)	26 (54%)		15 (47%)	10 (40%)	0.94
1	3 (20%)	8 (17%)		7 (22%)	7 (28%)	
2	0 (0%)	1 (2%)		2 (6%)	1 (12%)	
Unknown/Not yet	4 (37%)	13 (27%)		8 (25%)	7 (28%)	
**Best response 2^nd^ line chemotherapy**						
PD	6 (40%)	21 (44%)		15 (47%)	6 (24%)	0.33
SD	4 (27%)	13 (27%)		6 (19%)	9 (36%)	
PR	0	1 (2%)		3 (9%)	4 (16%)	
Unknown/Not yet	5 (33%)	13 (27%)		8 (25%)	6 (24%)	
**PD after 2^nd^ line chemotherapy**						
Yes	10 (66%)	33 (69%)		20 (62%)	20 (75%)	0.55
No	5 (34%)	15 (31%)		12 (38%)	5 (25%)	
**3^rd^ line therapy**						
Yes	7 (47%)	26 (54%)		13 (41%)	12 (48%)	0.72
No	2 (13%)	8 (17%)		9 (28%)	7 (28%)	
Unknown/Not yet	6 (40%)	14 (29%)		10 (31%)	6 (24%)	
**mOS (months)**	37.31	25.01		21.18	25.72	
**mPFS (months)**	8.62	9.54		10.19	10.39	

ECOG-PS, Eastern Cooperative Oncology Group—Performance Status; OS, Overall Survival; PFS, Progression Free Survival; PD, Progressive Disease; PR, Partial Response; SD, Stable Disease; MSI, Micro Satellite Instability; D-MMR, Deficient Mismatch Repair; P-MMR, Proficient Mismatch Repair.

In bold where statistically significant.

As for the entire cohort, 59/120 (49%) had partial response (PR), 42/120 (35%) had stable disease (SD), and 19/120 (16%) had progressive disease (PD) as the best response. Median PFS was 9.54 months (95% CI: 8.52–10.78), while median OS was 24.85 months (95% CI: 21.18–73.04). At the time of data cutoff, 104/120 (87%) have already progressed under first-line chemotherapy, and 85/120 (71%) have died already.

**Figure 2:**
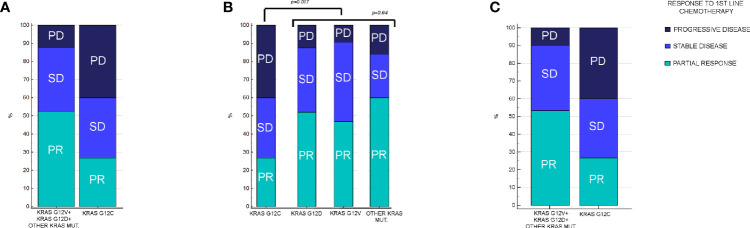
Response rates in patients enrolled in the analysis, stratified by KRAS mutation. **(A)** Response rates in KRAS G12C *vs* all other KRAS mutations combined. In KRAS G12C, PR: 27%, SD: 40%, and PD: 33%. In other KRAS mutations combined, PR: 52%, SD: 35%, and PD: 12% (p = 0.017). **(B)** Response rates between KRAS G12C *vs* G12D, G12V, and other KRAS mutations different from G12C, G12D, or G12V. In KRAS G12D, PR: 52%, SD: 35%, and PD: 13%. In KRAS G12V, PR: 47%, SD: 44%, and PD: 9%. In remaining KRAS mutations cohort, PR: 60%, SD: 24%, and PD: 16%. Difference in RR among KRAS G12V, G12D, and remaining mutations was not statistically significant (p = 0.64). **(C)** Response rates between KRAS G12C *vs* all other KRAS mutations combined after matching procedures. In other KRAS mutation cohort, PR: 56%, SD: 37%, and PD: 7% (p = 0.016).

In KRAS G12C mutated patients, 4/15 (27%) had PR, 6/15 (40%) had SD, and the remaining 5/15 (33%) had PD as the best response. In patients with other KRAS mutations, 55/105 (52%) had PR, 37/105 (35%) had SD, and the remaining 13/105 (12%) had PD as the best response. The difference in RR between patients who had KRAS G12C mutation *vs* other KRAS variants was statistically significant (p=0.017) (**Figure 2A**). On the other hand, response rates stratified by other KRAS mutation were not significantly different: in KRAS G12D mutated patients, 25/48 (52%) had PR, 17/48 (35%) had SD, and 6/48 (13%) had PD as the best response, while in KRAS G12V mutated patients, 15/32 (47%) had PR, 14/32 (44%) had SD, and 3/32 (9%) had PD. Finally, in patients with other KRAS mutations different from either G12C, G12D, or G12V, 15/25 (60%) had PR, 6/25 (24%) had SD, and the remaining 4/25 (16%) had PD. When comparing differences in RR among patients who had KRAS G12D, G12V, and other mutations different from G12C, no difference in RR was observed (p=0.64) (**Figure 2B**). In patients with exon3/4 mutations, 6/10 patients (60%) achieved PR, only 1 patient (10%) had SD, and the remaining 3 patients (30%) had PD as the best response. There was no difference in terms of activity compared with KRAS exon2 mutations besides KRAS G12C (p=0.08).

As for other variables used for univariate analysis, their association with differences in RR can be found in **Table 2**. As it can be seen, only age greater than 75 years old seemed to be associated with statistically significant differences in RR; however, multivariate analysis confirmed a statistically significant (p=0.0347) and independent role of G12C mutation as a predictor of worse RR, whereas age greater than 75 years old lost its statistical significance (p=0.0576).

**Table 2:** RR according to stratification factors.

Stratification Factor	PD	PR	SD	P value^α^
**KRAS mutation**				**0.0174**
G12C/Other	6 (40%)/13(13%)	4 (27%)/55(52%)	5 (33%)/37(35%)	
**Age at diagnosis**				**0.0354**
<75 years/≥75 years	14 (13%)/5(42%)	55 (51%)/4(33%)	39 (36%)/3(25%)
**Gender**				0.45
Male/Female	9 (13%)/10(20%)	37 (54%)/22(43%)	23 (33%)/19(37%)
**ECOG-PS**				0.16
0	11 (12%)	49 (55%)	30 (33%)
1	7 (25%)	10 (36%)	11 (40%)
2	1 (50%)	0	1 (50%)
**Sidedness**				0.80
Right/Left	8 (19%)/11(14%)	21 (49%)/38(49%)	14 (32%)/28(36%)
**Tumor grade**				0.98
1	1 (12%)	5 (63%)	2 (25%)
2	11 (14%)	40 (51%)	27 (35%)
3	2 (12%)	9 (57%)	5 31%)
**Mucinous histology**				0.10
Yes/No	6 (31%)/13(14%)	6 (31%)/50(54%)	7 (38%)/30(32%)
**Synchronous/Metachronous**				0.92
Synch/Meta	9 (14%)/10(18%)	31 (50%)/28(48%)	22 (36%)/20(34%)
**Liver metastases**				0.51
Yes/No	11 (13%)/8(22%)	42 (51%)/17(46%)	30 (36%)/12(32%)
**Lung metastases**				0.90
Yes/No	7 (16%)/12(15%)	20 (46%)/39(51%)	16 (38%)/26(34%)
**Peritoneal metastases**				0.92
Yes/No	5 (18%)/14(15%)	13 (46%)/46(50%)	10 (36%)/32(35%)
**Surgery (Primary tumor)**				0.81
Yes/No	14 (15%)/5(20%)	46 (49%)/13(50%)	34 (36%)/8(30%)
**Surgery (Resectable Metastases)**				0.56
Yes/No	3 (10%)/16(17%)	16 (59%)/43(47%)	9 (31%)/33(36%)
**Adjuvant chemotherapy**				0.65
Yes/No	9 (19%)/10(14%)	21 (46%)/38(51%)	16 (35%)/26(35%)
**IROX base**				0.72
IRI/OX	12 (18%)/7(13%)	32 (49%)/27(50%)	22 (33%)/20(37%)
**Total**	19 (16%)	59 (49%)	42 (35%)	

ECOG-PS, Eastern Cooperative Oncology Group—Performance Status; IROX base, Irinotecan- or Oxaliplatin-based first-line chemotherapy; PD, Progressive Disease; PR, Partial Response; SD, Stable Disease; CI, Confidence Interval.

^α^Statistically significant (P < 0.05).

In bold where statistically significant

**Figure 3:**
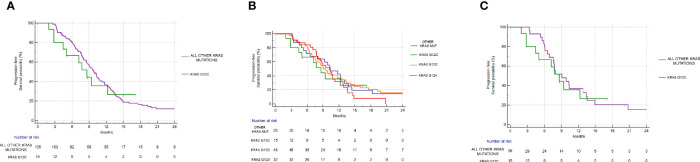
Progression free survival analysis. **(A)** PFS in KRAS G12C group *vs* all other KRAS mutations combined. mPFS: 8.62 *vs* 9.83 months, HR: 1.1, 95% CI: 0.55–2.20, p = 0.76. **(B)** PFS in KRAS G12C mutated *vs* KRAS G12D mutated *vs* KRAS G12V mutated *vs* other mutations. p = 0.83. **(C)** PFS in the KRAS G12C group *vs* all other KRAS mutations combined after matching procedures. mPFS: 8.62 *vs* 9.08 months, HR: 1.12, 95% CI: 0.52–2.41, p = 0.77.

**Figure 4:**
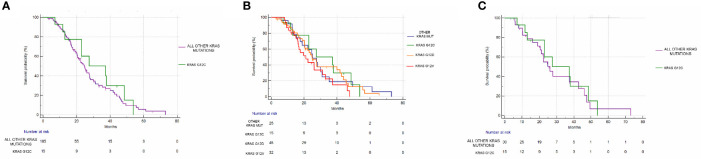
Overall survival analysis. **(A)** OS in the KRAS G12C group *vs* all other KRAS mutations combined. mOS: 37.31 *vs* 24.72 months, HR: 0.82, 95% CI: 0.44–1.52, p = 0.56. **(B)** OS in KRAS G12C mutated *vs* KRAS G12D mutated *vs* KRAS G12V mutated *vs* other mutations. p = 0.44. **(C)** OS in the KRAS G12C group *vs* all other KRAS mutations combined after matching procedures. mOS: 37.31 *vs* 26.09 months, HR: 0.86, 95% CI: 0.41–1.82, p = 0.70).

mPFS of the group of G12C mutated patients was 8.62 months *vs* 9.83 months of the group of patients with all other combined KRAS mutations (Hazard Ratio (HR): 1.1, 95% Confidence Interval (CI): 0.55–2.20, p=0.76) (**Figure 3A**). mOS in the G12C patients group was 37.31 months *vs* 24.72 months in patients with all other combined KRAS mutations (HR: 0.82, 95%CI: 0.44–1.52, p=0.56) (**Figure 4A**).

Even when considering the impact of each single KRAS mutation (G12C *vs* G12D *vs* G12V *vs* other mutations), no statistically significant differences in PFS (p=0.83) or OS (p=0.44) were seen. mPFS stratified by KRAS mutation was as follows: 8.62 months for G12C, 9.54 months for G12D, 10.19 months for G12V, and 10.39 months for other KRAS mutations (**Figure 3B**). mOS stratified by KRAS mutation was as follows: 37.31 months for G12C, 25.01 for G12D, 21.18 months for G12V, and 25.72 months for other KRAS mutations (**Figure 4B**).

Even after matching (**Supplementary Figures 1**, **2**), in patients with KRAS mutations different from G12C (30 patients), 17/30 (56%) had PR, 11/30 (37%) had SD, and 2/30 (7%) had PD as the best response. The difference in response rates remained statistically significant (p=0.016) (**Figure 2C**). As previously observed in the whole cohort, however, the HR for PFS of the matched cohort (HR: 1.24, 95%CI:0.58–2.67, p=0.55) (**Figure 3C**) as well as the HR for OS (HR:0.98, 95%0.47-2.08, p=0.97) (**Figure 4C**) remained not significantly different.

KRAS G12C *vs* other KRAS mutations were not associated with differences in sites of metastatic involvement (p=1 for liver metastases, p=0.56 for lung metastases, and p=0.51 for peritoneal metastases), sex (p=0.06), and ECOG PS (p=0.21). On the other hand, synchronous *vs* metachronous metastatic disease (p=0.039), age > 75 years (p=0.043), and mucinous histology (p=0.008) were more frequent in G12C mutated tumors compared with other KRAS mutations.

## Discussion

The landscape of KRAS mutations in colorectal cancer has widened considerably: first evidences pointing out at KRAS exon 2 mutations as predictors of primary resistance to anti-EGFR therapy in mCRC (1–3) have been joined with the results of further studies that have also proved the role of other mutational hotspots (in exons 3 and 4, respectively) (4).

Partly based on this fact, different KRAS mutations have always been considered as being the same in terms of response to other forms of treatment and in terms of prognostic value. This axiom has been challenged several times in the past, mainly in a series of retrospective analyses suggesting that different KRAS mutations might have different implications in clinical setting.

The old RASCAL II study, for example (13), suggested that in resected colorectal cancer patients stratified by KRAS mutational status, those harboring G12V mutations (8.6% of the analyzed population) were those with the higher risk of disease relapse, while other KRAS mutations did not have a prognostic impact. The difference between G12V *vs* other KRAS mutations was even more evident when considering high-risk patients with Dukes C stage (HR: 1.5) compared to Dukes B stage (HR: 1.15).

Currently, all KRAS variants are used as to identify those tumors who have primary resistance to anti-EGFR treatment: pooled analysis of patients enrolled in registrative trials of both Cetuximab and Panitumumab (14) showed that different KRAS variants might be associated with changes in treatment outcome among patients treated with chemotherapy combinations + anti-EGFR. In particular, G13D mutations seemed to behave differently from other KRAS variants: resistance against Panitumumab-based chemotherapy was observed, but data showed that Cetuximab-based chemotherapy might still improve survival. Primary resistance to both drugs was instead proven when assessing other KRAS mutations, and particularly G12A mutation was seen to be associated with worse survival outcomes in patients who received anti-EGFR therapy in combination with chemotherapy doublet. G13D mutations were also analyzed in other retrospective studies (15) confirming different outcomes in patients treated with Cetuximab monotherapy: patients with G13D mutated CRC had longer OS (mOS 7.6 *vs* 5.7 months, HR: 0.5, 95%CI: 0.31–0.81, p=0.005) and longer PFS (mPFS 4.0 *vs* 1.9 months, HR: 0.51, 95%CI: 0.32–0.81, p=0.004). *In vitro* and murine models suggested that G12V mutations were insensitive to Cetuximab, whereas G13D mutated cells were sensitive. Despite these findings, a prospective trial focused on the specific matter (16) failed to confirm these retrospective results: 51 G13D mutated mCRC patients were randomized to receive Cetuximab monotherapy or Cetuximab plus irinotecan combination, and there was no difference in the 6-month disease control rate between the two treatment arms. In particular, no responses were seen with single-agent cetuximab, thus disproving that KRAS G13D mutations might be associated with the clinical activity of Cetuximab (17).

These contradictory results, coupled with development of drugs against KRAS, have stemmed the interest in further research on the different behavior of KRAS variants in mCRC. Recently, two novel drugs directed against G12C KRAS mutation (Sotorasib and Adagrasib) (18) have been presented. Results of these drugs in mCRC patients have, however, been less favorable compared with other tumor types: response rates of mCRC patients treated with Sotorasib seem to be quite low compared with lung cancer patients. This fact has slowed down the development of these drugs in mCRC patients, as opposed to lung cancer patients. There is, however, a lack of data concerning different response rates with standard chemotherapy in patients who have been stratified by different KRAS variants. As to fill this gap in knowledge, the aim of our analysis was to assess differences in response rates of KRAS G12C mutated tumors compared to other KRAS mutations.

As expected, we were able to identify KRAS G12C mutation in 12% of the whole patient cohort: this percentage is comparable with other previous reports that showed percentages of G12C mutations ranging from 5% to 15%. As a matter of fact, we were able to identify also a relatively high percentage of patients harboring exon3/4 mutations (10/120 patients, 8%): this fact might have been due to the enhanced sensitivity of the tests used to ascertain KRAS mutational status.

Our study suggests that KRAS G12C mutated tumors might have reduced sensitivity to standard chemotherapy doublet + Bevacizumab. Indeed, albeit response rates observed in KRAS mutated patients with chemotherapy doublet + Bevacizumab is usually around 45–50% (19), as also confirmed in our group of patients who did not have KRAS G12C mutation, KRAS G12C mutated tumor response rates were only 27%. This was maintained also when matching for other prognostic factors. On the other hand, all other KRAS mutations (including also those in exon3/4) had similar outcomes in terms of RR, PFS, and OS.

We were not able to confirm a potential prognostic role of KRAS G12C mutation when PFS was assessed: mPFS was slightly worse (8.63 months) in KRAS G12C patients as opposed to those who harbored different KRAS mutations, but the difference was not statistically significant. In addition to that, no differences in overall survival were evident, and this fact was even confirmed when matching for all other prognostic factors: even though a series of papers have suggested a prognostic role of KRAS G12C mutation also in terms of different PFS and OS, all these analyses lack proper adjustment for confounding factors.

Indeed, looking at other prognostic factors that might have lessened the impact of KRAS G12C mutation both on PFS and OS, it can be observed that the cohort of our study is well balanced for all stratification factors that are usually considered when assessing survival outcomes. Other studies have not performed the same: George et al. (20), for example, have reported that KRAS G12C mutations might be associated with statistically significant differences in OS. Differently from our study, those authors did not report any kind of matching for other relevant prognostic factors (ECOG PS, tumor sidedness, surgery of metastatic sites with radical intent, etc.), and they did not also report which kind of palliative chemotherapy regimen those patients had received, thus preventing any other comparison with our study. Recently, Chida et al. (21) have also reported that KRAS G12C mutation might be associated with worse prognosis in patients affected by mCRC treated with first-line chemotherapy. It must be noticed, however, that the authors have enrolled a rather heterogenous group of patients, treated with different chemotherapy combinations (monotherapy, doublets, and triplets), and there was a significant proportion of patients who did not receive first-line treatment with Bevacizumab. It should also be considered that this study was conducted in Japan, and a large number of patients received also S-1-based chemotherapy combinations: since this drug is mainly used in Eastern countries due to different toxicity and activity when compared to Western countries, we believe that this study cannot be used for comparison with our results.

To our knowledge, our study is the first that has focused specifically on assessing KRAS G12C impact on response rates during first-line therapy, in addition to its role as a prognostic factor for OS and PFS. We believe that the lack of responses that was seen in our group of patients should be discussed further, as it represents both a therapeutic challenge and a matter to be investigated upon also from a biological point of view.

Indeed, despite increased rate of progressive disease during first-line therapy and despite lower rates of partial responses, differences in survival outcomes for these patients were rather negligible. This fact suggests that these tumors might have primary resistance to chemotherapy but also relatively slower clinical behavior compared with other tumors that have different KRAS mutations. Some preclinical data have suggested that G12C mutated colorectal cancer lines have metabolic characteristics that lead to evasion of apoptosis rather than increased growth rate (22).

As a matter of fact, our study also suggests that response rates might be lower in KRAS G12C mutated patients regardless of which type of chemotherapy doublet + Bevacizumab has been used. This fact should renew the interest in further research on this specific group of patients, either suggesting that “aggressive” chemotherapy combinations that have higher likelihood of response should be used (as in the case of FOLFOXIRI+Bevacizumab) or with the introduction of specific G12C inhibitors also in these patients. Indeed, the failure in being able to obtain radiological responses in patients who were treated with these drugs such as in the CODEBREAK-100 trial might not be due to lack of effectiveness of the drug but rather to different metabolism of the tumor itself that might reduce the likelihood of being able to see quick changes in the tumor mass. It is then hoped that new trials focused on combining KRAS G12C inhibitors with standard chemotherapy doublet + Bevacizumab for mCRC patients are conducted in the near future.

## Data Availability Statement

The original contributions presented in the study are included in the article/**Supplementary Material**. Data concerning this analysis is freely available upon reasonable request directed to the corresponding author.

## Ethics Statement

The studies involving human participants were reviewed and approved by Comitato Etico Regione Marche—C.E.R.M. Written informed consent for participation was not required for this study in accordance with the national legislation and the institutional requirements.

## Author Contributions

RG and MS contributed to study concept and design. AL, CC, SC, PZ, EG, FP, AM, AP, MP, and AB contributed to acquisition, analysis, or interpretation of data. RB, RG, MS, AL, SC, and AB contributed to critical revision of the manuscript for important intellectual content. RG contributed to statistical analysis. MS, RB, and AB contributed to administrative, technical, or material support. RG, AL, CC, FP, PZ, AP, MP, and SC contributed to study supervision. All authors contributed to the article and approved the submitted version.

## Conflict of Interest

The authors declare that the research was conducted in the absence of any commercial or financial relationships that could be construed as a potential conflict of interest.

## Publisher’s Note

All claims expressed in this article are solely those of the authors and do not necessarily represent those of their affiliated organizations, or those of the publisher, the editors and the reviewers. Any product that may be evaluated in this article, or claim that may be made by its manufacturer, is not guaranteed or endorsed by the publisher.

## Abbreviations

mCRC, metastatic colon rectal cancer; NSCLC, non-small cell lung cancer; RR, response rate; 95%CI, 95% confidence interval; HR, Hazard Ratio; EGFR, Epidermal Growth Factor Receptor; KRAS, Kirsten Rat Sarcoma Oncogene Viral Homologue; PFS/mPFS, progression free survival/median progression free survival; OS/mOS, overall survival/median progression free survival; ECOG PS, Eastern Operative Oncology Group Performance Status.
