# Supplementary material for: Retrospective Comparative Analysis of KRAS G12C vs. Other KRAS Mutations in mCRC Patients Treated With First-Line Chemotherapy Doublet + Bevacizumab
Source: Front Oncol. 2021 Sep 30;11:736104. doi: 10.3389/fonc.2021.736104 (PMC8514824; doi:10.3389/fonc.2021.736104)

Supplementary Material

## Supplementary Figures

**
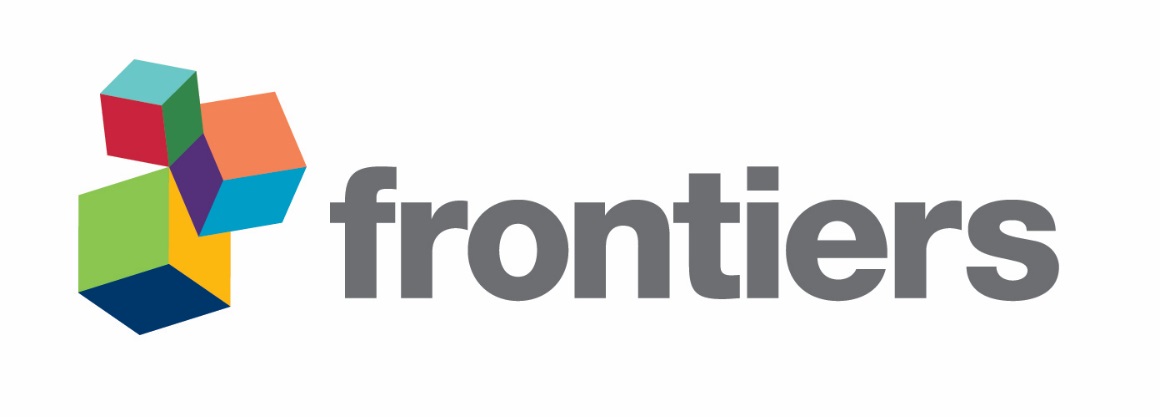
**

Supplementary Figure 1: Propensity score matched results. JITTER plot.
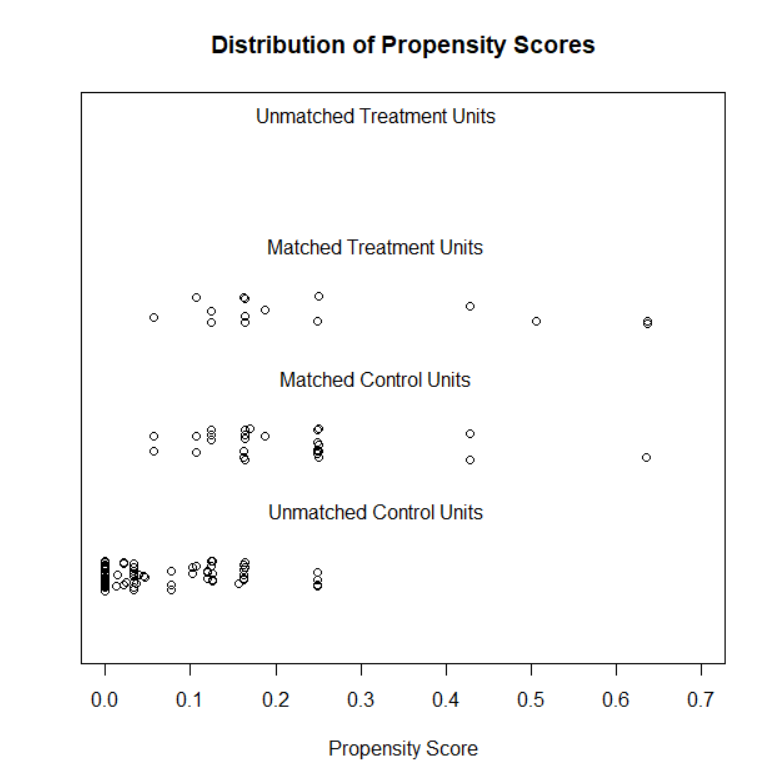


Supplementary Figure 2: Propensity score matched results. Histogram.


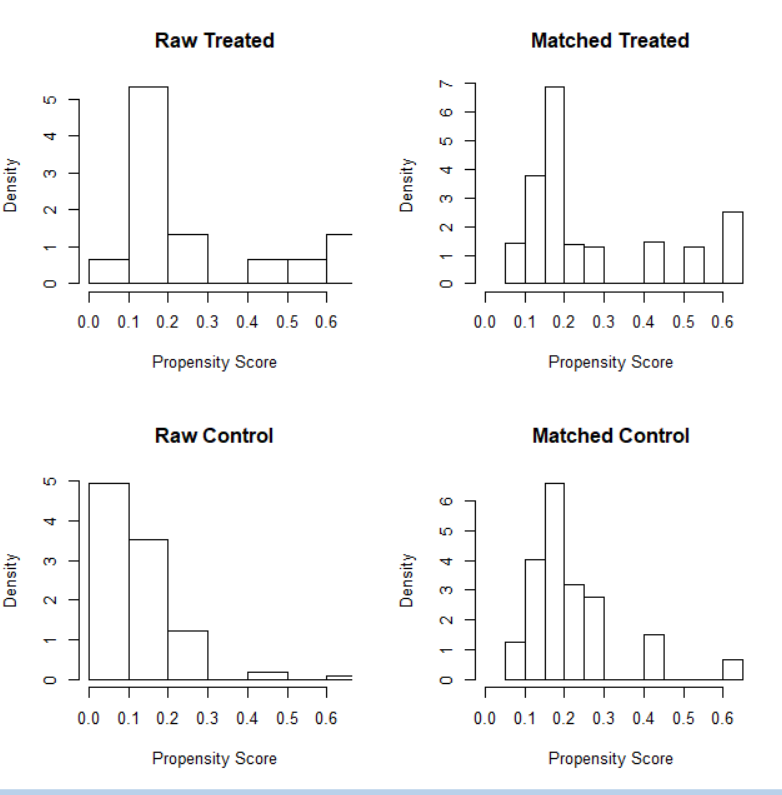

Supplement: Supplementary file 1 [file DataSheet_1.docx]
